# Patients and family caregivers report high treatment expectations during palliative chemotherapy: a longitudinal prospective study

**DOI:** 10.1186/s12904-021-00731-4

**Published:** 2021-02-26

**Authors:** Tine Ikander, Stefan Starup Jeppesen, Olfred Hansen, Mette Raunkiær, Karin Brochstedt Dieperink

**Affiliations:** 1 Department of Oncology, Academy of Geriatric Cancer Research (AgeCare), Odense University Hospital, J.B. Winsløws Vej 4, 5000 Odense, Denmark; 2 REHPA, The Danish Knowledge Centre for Rehabilitation and Palliative Care, Odense University Hospital and University of Southern Denmark, Nyborg, Denmark; 3 Department of Clinical Research, University of Southern, Family Focused Healthcare Research Centre (FaCe), Odense, Denmark; 4 Department of Clinical Research, University of Southern, Odense, Denmark; 5 OPEN, Open Patient data Explorative Network, Odense University Hospital, Region of Southern, Odense, Denmark

**Keywords:** Quality of life, Lung neoplasms, Longitudinal studies, Palliative care, Family caregivers

## Abstract

**Background:**

When discussing treatment options and future care, it is important to understand the expectations of patients and family caregivers related to palliative chemotherapy and to identify patterns in patients’ quality of life. The study aims were to evaluate differences in treatment expectations and quality of life between patients with thoracic cancer (non-small-cell lung cancer, small-cell lung cancer and mesothelioma) who were < 70 and ≥ 70 years of age and receiving palliative chemotherapy and to assess family caregivers’ treatment expectations.

**Methods:**

A prospective longitudinal study included patients with thoracic cancer receiving outpatient palliative chemotherapy at a university hospital in Denmark and their family caregivers. Patients’ treatment expectations and quality of life were assessed three times during treatment with a survey of treatment expectations and the Functional Assessment of Cancer Therapy – General questionnaire. Family caregivers’ treatment expectations were assessed once.

**Results:**

A total of 48 patients and 36 family caregivers participated between 2018 and 2019. No statistically significant age-related differences in treatment expectations and quality of life were identified. 28% of patients aged < 70 years and 7% of those aged ≥70 years expected a cure. Among family caregivers, 36% expected a cure. Across both age groups, mean total quality of life scores significantly decreased from 73.2 at first palliative chemotherapy cycle to 70.5 at third cycle (*p =* 0.02). No meaningful changes were found in quality of life within either age group. A subgroup analysis found no significant between-group differences in quality of life. Mean physical well-being score for all patients decreased from 20.3 at first cycle to 18.4 at third cycle (*p =* 0.03) and mean emotional well-being score decreased from 15.4 at first cycle to 14.6 at third cycle (*p =* 0.04).

**Conclusion:**

This study emphasizes the importance of initiating conversations about treatment expectations and paying attention to expectations that may differ by the age of the patient and between patients and family caregivers. Addressing treatment expectations among patients and family caregivers and monitoring quality of life among patients is important in clinical practice.

**Supplementary Information:**

The online version contains supplementary material available at 10.1186/s12904-021-00731-4.

## Background

Patients with thoracic cancer are often diagnosed late in the course of disease and consequently have a poor prognosis [1, 2]. However, during the past decade, new antineoplastic treatment modalities have altered the disease trajectory of thoracic cancer patients. Many patients are offered palliative chemotherapy to prolong life and improve quality of life (QoL) near the end of life [3–5]. One-year survival for patients in Denmark with newly diagnosed lung cancer receiving palliative treatment is 32% [6]. To make sound treatment decisions, it is important to understand patients’ and family caregivers’ personal values and expectations about palliative treatment and to assess patients’ QoL [7, 8]. Because family caregivers often provide primary support when patients when difficult treatment decisions must be made, it is important that patients and family caregivers share an understanding of the prognosis. Patients with advanced cancer may have an unrealistic understanding of treatment, believing or hoping that palliative chemotherapy may lead to a cure [9, 10]. A systematic review concluded that very little knowledge exists about the expectations of patients with thoracic cancer about palliative chemotherapy [11]. QoL is widely accepted as a clinically meaningful endpoint to assess the benefits of chemotherapy. Previous studies investigating longitudinal assessments of QoL among patients with primarily non-small-cell lung cancer (NSCLC) receiving palliative chemotherapy have reported no significant decrease in QoL [12–16].

Knowledge about how treatment expectations and QoL differ between younger and older patients is important to making treatment decisions. We found no previous studies exploring the impact of age on treatment expectations. However, older patients are more likely to have serious side effects that may affect QoL during palliative treatment [17]. A randomized controlled trial investigating the effect of vinorelbine compared to best supportive care among patients diagnosed with NSCLC who were older than 70 years found that vinorelbine-treated patients had worse treatment-related outcomes, i.e., constipation, nausea and hair loss, but better cancer-specific outcomes, i.e., pain and dyspnoea [18]. However, to the best of our knowledge, only a few studies have evaluated age-related QoL among patients being treated for NSCLC, small-cell-lung cancer (SCLC), or mesothelioma. Wintner et al. followed 220 patients with NSCLC and SCLC from adjuvant to palliative chemotherapy, finding no difference in QoL between patients aged < 70 and ≥ 70 years, except for decreased physical functioning over time among those aged < 70 years [12]. Hensing et al. also reported no difference in QoL between patients aged < 70 and ≥ 70 years who were receiving first-line treatment with curative intent for advanced NSCLC [19]. We found no published studies investigating the influence of age on QoL among patients receiving only palliative chemotherapy.

The study aim was to examine differences in treatment expectations and QoL among patients with thoracic cancer aged < 70 and ≥ 70 years who were receiving palliative chemotherapy and to assess family caregivers’ expectations for palliative chemotherapy. We hypothesized that patients aged ≥70 years would have worse QoL and lower treatment expectations than younger patients.

## Methods

In a prospective, longitudinal study, patients with thoracic cancer and their family caregivers were recruited from the outpatient oncology clinic at Odense University Hospital in March 2018–February 2019. The cut point for age of 70 years is commonly used in geriatric oncology [20]. Nurses and oncologists at the clinic provide palliative care but do not specialize in it. The Danish Data Protection Agency (study ref. no. 18/60988) approved the study. Approval by the local ethics committee was not required (project ID: S-20172000-90). Verbal and written informed consent were obtained from all study participants. Study data were collected and managed using REDCap electronic data capture [21, 22].

### Participants and procedures

The first author screened consecutive patients for study eligibility. Inclusion criteria were: 1) a diagnosed NSCLC, SCLC or mesothelioma, 2) initiation of first- to fifth-line palliative chemotherapy, 3) ability to read and speak Danish, and 4) aged ≥18 years.

Family caregiver inclusion criteria were: 1) providing care for patients undergoing palliative chemotherapy, 2) attending the first clinical appointment, 3) able to read and speak Danish, and 4) aged ≥18 years. All participating patients received first- to fifth-line treatment; the effect of later line treatment generally decreases, with poor prognosis.

### Measurements

Patients’ treatment expectations and QoL were assessed by questionnaire before the first cycle of palliative chemotherapy and at the second (3 weeks later) and third (6 weeks later) cycles. These time points were selected because palliative treatment was re-evaluated after two cycles, when patients had a CT scan and a subsequent appointment with the physician to make further treatment decisions. Family caregivers’ expectations for palliative treatment were assessed only at the first chemotherapy cycle. In addition, all participants completed questions about sociodemographic characteristics. Participants completed questionnaires during the clinic visit or at home.

Treatment expectations were assessed with a single item with four response options: ‘reduced pain and discomfort’, ‘prolongation of life’, ‘cure’ and ‘don’t know’ [Additional file 1]. Participants could select more than one response option. The item was based on earlier studies and pilot tested using cognitive interviews [9, 10, 23].

QoL data was assessed with a licensed version of The Functional Assessment of Cancer Therapy – General Questionnaire (FACT-G, version 4) [24]. It consists of 28 questions covering four domains: physical well-being (7 items; domain score range, 0–28), social/family well-being (7 items; domain score range, 0–28), emotional well-being (6 items; domain score range, 0–24), and functional well-being (7 items; domain score range, 0–28). The range of possible total FACT-G scores is 0–108, with higher scores indicating better QoL. Clinical data were collected from medical records.

### Statistical analysis

Sociodemographic and clinical characteristics of patients and family caregivers were described by medians for continuous variables and proportions for categorical variables. The statistical significance of differences between patient age groups was assessed with the Wilcoxon rank sum test and Fisher’s exact test. To detect any potential effect of attrition on the outcomes of interest, we also calculated completion rates as the number of completed questionnaires divided by the number of expected questionnaires.

Changes over time from baseline in total and domain FACT-G scores were evaluated with one-way ANOVA with the Greenhouse-Geisser correction and a QoL and time interaction term. Repeated measures one-way ANOVA was also used for sub analyses of FACT-G domains. Changes of ≥5 points in FACT-G total scores and ≥ 2 points in domain scores were considered clinically meaningful [25]. The statistical significance of changes in patients’ treatment expectations from baseline to the second and third cycles of chemotherapy was assessed with Student’s t-test. Cohen’s kappa coefficient assessed agreement between patients’ and family caregivers’ treatment expectations, with a value of 0 indicating non-agreement and a value of 1 indicating perfect agreement [26]. Missing items in subscales were handled according to the FACIT Administration and Scoring Guidelines [27]; the subscale score was multiplied by the number of items in the subscale, then divided by the number of items answered. A *p* value < .05 was considered statistically significant. All analyses were conducted using STATA 15 [28].

## Results

### Demographic and clinical characteristics

Of 58 invited patients, *n* = 48 (83%) and *n* = 36 of their family caregivers consented to participate in the study during the course of palliative chemotherapy. Planned treatment regimens for participating patients included docetaxel, emetrexed, etoposid, gemcitabin, carboplatin, vinorelbine, or topotecan as monotherapy or in combination chemotherapy. Most patient participants were male (65%), and median age of all participants was 66 years (range, 49–81) (Table 1). Compared to those in the older group, more patients in the younger group had a better performance status at baseline (*p* = 0.02) and were ex-smokers (*p* = 0.01) and fewer were diagnosed with mesothelioma (*p* = 0.002). Of family caregivers, 81% were female. Median age of all family caregivers was 62.5 years (range, 19–74). Family caregivers were patients’ spouses (75%), partners (8%), or children (17%). Tables 1 and 2 contain complete participant characteristics.

**Table 1:** Patient characteristics in a study with patients diagnosed thoracic cancer in palliative chemotherapy

	All patients (*n* = 48)	< 70 years (*n* = 30)	≥70 years (*n* = 18)	
Variable	n(%)	n(%)	n(%)	*P*^*1*^
Sex				0.36
Female	17(35)	9(30)	8(44)	
Male	31(65)	21(70)	10(56)	
Age (years)				0.81
Median	66	62	75	
Range	49–81	49–68	70–81	
Malignant diagnosis				0.002
NSCLC	31(65)	23(77)	8(44)	
SCLC	8(17)	6(20)	2(11)	
Mesothelioma	9(19)	1(3)	8(44)	
Palliative chemotherapy				0.82
1st line	5(10)	4(13)	1(6)	
2nd line	25(52)	15(50)	10(56)	
3rd line	15(31)	9(30)	6(33)	
4th line	2(4)	1(3)	1(6)	
5th line	1(2)	1(3)	0(0)	
Relation				0.45
Living alone	8(17)	4(13)	4(22)	
Living with spouse/possibly children	40(83)	26(87)	14(78)	
Education				0.79
(Basic) Less than 10 years	40(19)	11(37)	8(44)	
Youth (high school)	21(44)	13(43)	8(44)	
Medium (profession)	8(17)	6(20)	2(11)	
Higher (university)	0	0	0	
Smoking status				0.01
Smoker	16(33)	7(23)	9(50)	
Ex. smoker	24(50)	20(67)	4(22)	
Never smoked	8(17)	3(10)	5(28)	
Performance status				0.06
0–1	39(81)	17(90)	12(67)	
2	9(19)	3(10)	6(33)	

^1^ *P* values were derived from Fisher’s exact test and Wilcoxon rank sum test

**Table 2:** Characteristics of family caregivers for patients diagnosed with thoracic cancer receiving palliative chemotherapy

Variable	n(%)
Sex	
Female	29(81)
Male	7(19)
Age (years)^a^	
Median	62.5
Range	19–74
Unknown	2
Relation	
Spouse	27(75)
Partner	3(8)
Child	6(17)

^a^ Missing data for two family caregivers

### Completion rates

Completion rates for patients’ treatment expectation questionnaires during the second and third chemotherapy cycles were 90 and 83%, respectively. FACT-G completion rates during second and third cycles were 92 and 83%, respectively.

### Treatment expectations

**Fig. 1:**
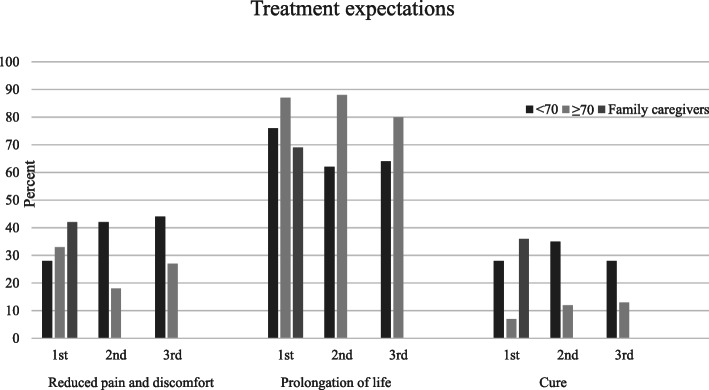
Agreement between patient and family caregiver treatment expectations

No statistically significant differences in treatment expectations were found between patients aged < 70 years and those aged ≥70 years. At the first cycle, 28% of patients in the younger group expected a cure, compared to 7% in the older group, but this difference did not reach statistical significance (*p* = 0.1), nor did expectations change across the second and third cycles. There was no difference in treatment expectations regarding reduced pain and discomfort and prolongation of life (Table 3 and Fig. 1). Eighteen (38%) patients did not change their expectations of palliative chemotherapy after the first treatment.

**Table 3:** Patient treatment expectations for palliative chemotherapy

		< 70 years			≥70 years		
Patient expectations*	No.	Total no.	%	No.	Total no.	%	P^**^
Reduced pain and discomfort							
1st cycle	8	21	28	5	10	33	.7
2nd cycle	11	26	42	3	17	18	.1
3st cycle	11	25	44	4	15	27	.3
Prolongation of life							
1st cycle	22	29	76	13	13	87	.7
2nd cycle	16	26	62	15	18	88	.08
3rd cycle	16	25	64	12	15	80	.5
Cure							
1st cycle	8	29	28	1	15	7	.1
2nd cycle	9	26	35	2	17	12	.2
3rd cycle	7	25	28	2	15	13	.4

^*^Patients could choose more than one answer

^**^P values were derived from Fisher’s exact test

Among family caregivers, *n* = 13 (36%) expected a cure, *n* = 25 (69%) expected prolongation of life, and *n* = 15 (42%) expected reduced pain and discomfort for the patient (Table 4). Of family caregivers who expected a cure, *n* = 12 (92%) provided care for patients younger than 70 years. Agreement on treatment expectations between patient and family caregivers was 66.67% (Cohen’s kappa, 0.20) for reduced pain and discomfort, 58.33% (Cohen’s kappa, 0.15) for prolongation of life, and 79.17% (Cohen’s kappa, 0.42) for cure.

**Table 4:** Family caregiver treatment expectations for palliative chemotherapy

Expectations of family caregivers *n* = 36^a^	No.	%
Reduced pain and discomfort	15	42
Prolongation of life	25	69
Cure	13	36

^a^Family caregivers could choose more than one answer

### Quality of life

**Fig. 2:**
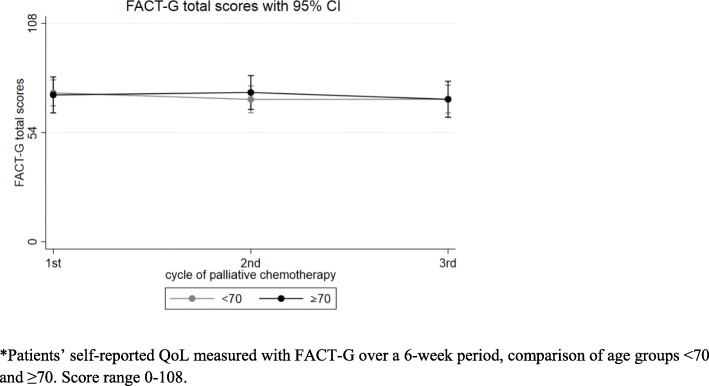
Functional Assessment of Cancer Therapy-General (FACT-G) total scores* in a study with thoracic cancer patients in palliative chemotherapy

No statistically significant difference was observed for the interaction term of QoL and time (*p* = 0.83) between patient groups. Younger patients reported better QoL at the first cycle than did older patients (mean, 73.6 vs 72.6). QoL in the two age groups did not differ at the third line of palliative chemotherapy (Fig. 2). Overall mean QoL scores significantly decreased over the course of palliative chemotherapy among all patients from 73.2 at first cycle to 70.5 at third cycle (*p* = 0.02). However, this decrease was not clinically meaningful [25].

In QoL domain analyses, no statistically significant differences were found between age groups. Mean physical well-being domain scores at the three cycles among younger patients were 20.2, 19.6, and 18.1, compared to 20.5, 19.9, and 19.1 among older patients (*p* = 0.90). However, the decline in mean physical well-being score among patients aged < 70 years was 2.1, indicating clinical significance. A subgroup analysis of 36 patients who completed the questionnaire at all three visits confirmed this finding. In addition, mean physical well-being scores among all patients significantly declined over time from 20.3 to 19.7 and 18.4 (*p* = 0.03), nearing clinical significance.

Mean social/family well-being domain scores at the three cycles among younger patients were 20.5, 19.5, 20.5, compared to 19.9, 20.7, and 19.7 among older patients (*p* = 0.19). The mean domain score for all patients did not change over time.

Mean emotional well-being domain scores at the three cycles among younger patients were 15.4, 15.0, and 14.8, compared to 15.5, 15.8, and 14.7 among older patients (*p* = 0.81). However, the mean emotional well-being domain score among all patients significantly decreased over time, from 15.4 to 15.2 and 14.6 (*p =* 0.04). Mean functional well-being domain scores at the three cycles among younger patients were 17.5, 16.3, and 17.2, compared to 16.5, 17.8, and 17.1 among older patients (*p =* 0.59). The mean functional well-being domain score among all patients remained stable over the three cycles at 17.2, 16.8, and 17.1 (*p =* 0.45). Subgroup analyses details are available in Additional file 2.

## Discussion

### Treatment expectations

Treatment expectations did not differ between patients aged < 70 and ≥ 70 years. However, a significant minority of patients (*n* = 9, 21%) and their family caregivers (*n* = 13, 36%) believed that palliative treatment would cure the cancer at the first cycle of chemotherapy. Previous studies have demonstrated that patients diagnosed with incurable cancer expected that palliative chemotherapy could lead to the cancer being cured [10, 29, 30]. In a US study by Temel et al., of 74 patients with NSCLC who received palliative chemotherapy or radiotherapy, 30% expected a cure [9]. This finding is similar to the younger patients in our study, of whom 28–35% expected a cure from treatment. A survey study from the US also demonstrated that younger patients aged < 57 years were significantly more likely to hope for a cure [31]. There may be many reasons why younger patients have higher expectations of palliative chemotherapy. High expectations may arise from insufficient information from physicians, as discussed by Nowicki et al. [30]; especially when the prognosis is poor, physicians may find it difficult to communicate bad news, which also could be the case in our study. Additionally, patients may misunderstand physicians when they say that the cancer is responding to the treatment [11]. Patients may believe that responding to the treatment means they are being cured of cancer. None of the patients in our study were highly educated; it is important to acknowledge that patients with thoracic cancer may generally have low health literacy, which can affect their capacity to understand basic information, such as prognostic information [32]. In patients aged ≥70 years, the proportion expecting a cure increased from 7 to 13% from first to third cycle, which may indicate that patients receiving chemotherapy achieved better symptom control.

A study group [33] developed a decision tool in a pilot study in Canada to help inform patients with lung cancer about prognosis and treatment options. The tool presented information about survival and effects on QoL, along with an explicit statement that the chemotherapy was not given with a curative intent. Despite the effort to create an optimised information tool, all patients retained unrealistic hopes of a cure or, perhaps, of a miracle [33]. This suggests that physicians may provide information but patients may not assimilate it, which could be a coping mechanism [33]. Moreover, older patients may be more likely to accept poor prognoses because they are less likely to have dependent children and will lose fewer years of their working lives [31]. However, in our study, no significant difference in expectations between younger and older patients was observed, even though treatment expectations among younger patients were high; this could be due to the small number of patients in the sample. Although we found no significant age-related differences, previous international studies suggest there may be age-related variations in treatment expectations [9, 31]. When initiating palliative treatment, it may be important to incorporate conversations about expectations, paying particular attention to potential age-related differences and differences between patient and family caregivers.

In our study, 36% of family caregivers expected a cure. One possible explanation for treatment expectations among family caregivers is the fact that the questionnaire was administered when patients were starting the first cycle of chemotherapy. Some family caregivers may not have been present at the consultation during which patients were told their cancer had progressed. Another reasonable explanation for high treatment expectations is that 81% of patients included in the study had good performance status. However, a 2019 study revealed discordance in beliefs about curability in 52% of caregiver-oncologist dyads, indicating that family caregivers often have unrealistic treatment expectations [34]. Our study also demonstrated that 42% of family caregivers expected reduced pain and discomfort at the first cycle, compared to 28% of patients. It may be difficult for a family caregiver to see a close relative who is affected by an incurable cancer suffer near the end of life. Family caregivers are often the closest source of support during cancer treatment [35], and differences in expectations between patients and caregivers may prevent them from making timely decisions about treatment and future care. Physicians and nurses must assess and, as needed, adjust patient and family caregiver expectations. It is important to note that the expectations and experiences of family caregivers during palliative chemotherapy have not been adequately examined, and further studies with more participants are needed.

### Quality of life

No age-related difference was observed in the QoL and time interaction term, consistent with other studies [12, 19]. However, overall QoL decreased over time for all patients to a statistically significant but not clinically meaningful degree [25]. In subgroup analyses, physical well-being and emotional well-being declined significantly among all patients. Wintner et al. [12] found similar results for QoL during different treatment lines in patients aged < 70 and ≥ 70 years with NSCLC and SCLC, as measured with the European Organisation for Research and Treatment of Cancer Quality-of-life Questionnaire Core 30. The same study also documented a decline in the physical functioning QoL domain among younger patients [12]. Although, unlike Wintner et al., we found no significant difference between age groups in physical well-being, we observed a clinically meaningful but not statistically significant decline in the physical well-being domain among younger patients, whose baseline performance status was better than that of older ones. Wintner et al. also found decreased physical functioning among patients aged ≥70 years in third or later lines of treatment [12]. We were unable to replicate this finding due to the relatively small number of patients in the older age group receiving third-line or later palliative chemotherapy.

It may be difficult to assess the relative influence on QoL of palliative treatment or other factors like comorbidity and high symptom burden. The challenge is to identify patients who truly benefit from palliative chemotherapy and find the right time to discontinue it. Routine assessment of QoL and expectations at baseline and during palliative chemotherapy may increase awareness among physicians and nurses of patients’ and family caregivers’ perspectives on treatment and understanding of disease. QoL assessment provides important information about symptoms arising from treatment and insight into other aspects of physical, emotional, social/family, and functional well-being. Using QoL assessment systematically during palliative treatment may provide guidance about the timing of conversations about treatment decisions.

### Strengths and limitations

This study has several limitations that deserve mention. Relatively few patients and family caregivers were included despite an 11-month inclusion period. The inclusion process was very time-consuming because many patients’ chemotherapy was postponed due to admissions, side effects, or other cancer-related problems. The second and third cycles of palliative chemotherapy were postponed for 13 and 8 patients, respectively. In addition, six patients died during the study period. This demonstrates the complexity of collecting data in a real-life palliative care setting. Another limitation is the interval of 3 weeks between QoL questionnaires, which may account for the lack of significant between-group findings. Nevertheless, among patients receiving palliative chemotherapy with a poor prognosis, assessment of QoL over shorter time intervals is likely necessary. Despite these challenges, the participant completion rate was an acceptable 83%. Kristensen and colleagues had an equivalent completion rate in a longitudinal study among patients affected by lung cancer receiving chemotherapy [13].

An additional limitation is that no validated tool exists with which to assess treatment expectations among patients with advanced cancer. Our single item addressing expectations was developed from earlier studies [9, 10] and pilot tested with cognitive interviews before use [21]. Furthermore, the exploratory nature of our study was intended to provide insight into the intersection of age, expectations, and QoL. Future studies should include larger patient cohorts to provide statistically rigorous information about when to discuss withdrawal from chemotherapy with patients. Future in-depth studies should investigate collaboration between patients, caregivers, and professionals about treatment decisions and its relationship to treatment expectations and end-of-life discussions*.* Furthermore, it may be beneficial to adjust patients’ and family caregivers’ expectations and initiate shared decision making in end-of-life treatment.

Following a representative sample of patients with thoracic cancer closely over time, we have new insight into the expectations of Danish patients with thoracic cancer and their family caregivers and QoL during palliative chemotherapy.

## Conclusion

No significant differences were observed in treatment expectations and QoL between patients aged < 70 and ≥ 70 years. Overall QoL decreased significantly over time, especially in the domains of physical and emotional well-being. A higher percentage of younger patients expected a cure than older ones, and more than a third of family caregivers expected a cure from palliative treatment. This emphasizes the importance of initiating discussions about treatment expectations and attending carefully to different understandings of treatment across ages and between patients and their family caregivers. Assessing expectations among patients treated with palliative chemotherapy and their family caregivers and monitoring quality of life among patients is important in clinical practice.

## Supplementary Information

**Additional file 1.**

**Additional file 2.**

## Data Availability

The dataset/analyses used during the current study are available from the corresponding author on reasonable request.

## Abbreviations

QoL: Quality of life
FACT-G: The functional assessment of cancer therapy – general questionnaire
NSCLC: Non-small-cell lung cancer
SCLC: Small-cell lung cancer
