# Supplementary material for: Patients and family caregivers report high treatment expectations during palliative chemotherapy: a longitudinal prospective study
Source: BMC Palliat Care. 2021 Feb 26;20:37. doi: 10.1186/s12904-021-00731-4 (PMC7912463; doi:10.1186/s12904-021-00731-4)
Supplement: Supplementary file 1 — Additional file 1. [file 12904_2021_731_MOESM1_ESM.docx]

| **After talking to your doctor about the cancer treatment, what are your primary expectations of the treatment you will receive today?**  *(More answers are allowed)* | |
| --- | --- |
| **Reduced pain and discomfort** | **Prolongation of life** |
| **Cure** | **Don’t know** |

**Additional file 1: Survey on treatment expectations**
