# Supplementary material for: Patients and family caregivers report high treatment expectations during palliative chemotherapy: a longitudinal prospective study
Source: BMC Palliat Care. 2021 Feb 26;20:37. doi: 10.1186/s12904-021-00731-4 (PMC7912463; doi:10.1186/s12904-021-00731-4)
Supplement: Supplementary file 2 — Additional file 2. [file 12904_2021_731_MOESM2_ESM.docx]

**Additional file 2: Sub-group analysis**

**
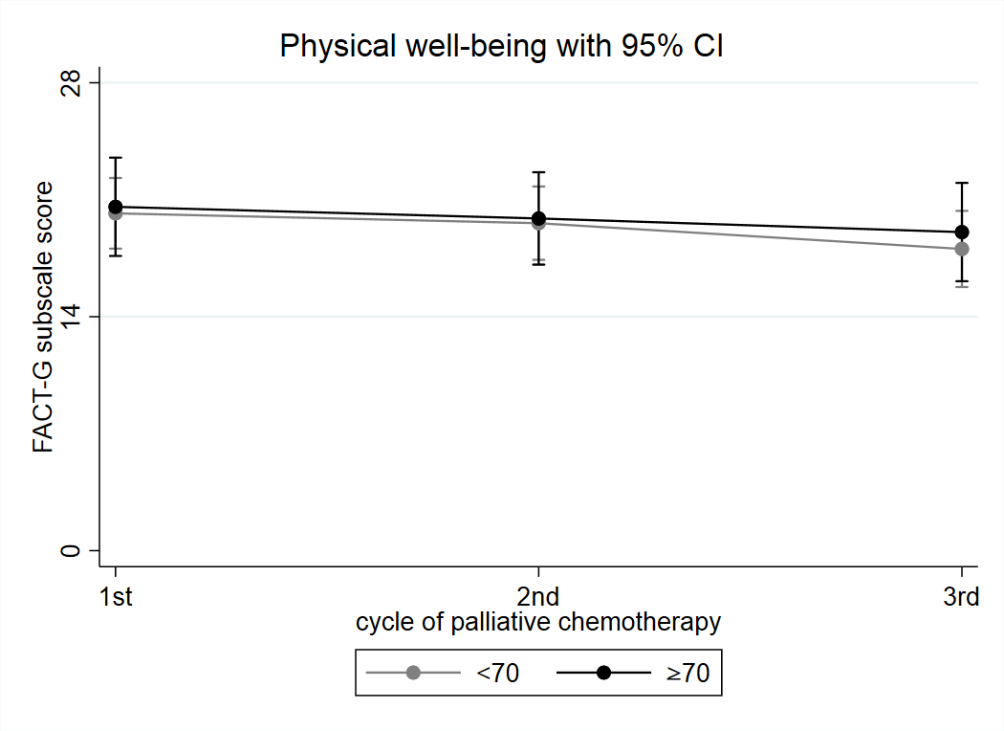
**

*Patients’ self-reported QoL measured with FACT-G over a 6-week period, comparison of age groups <70 and ≥70. Score range 0-28.

**
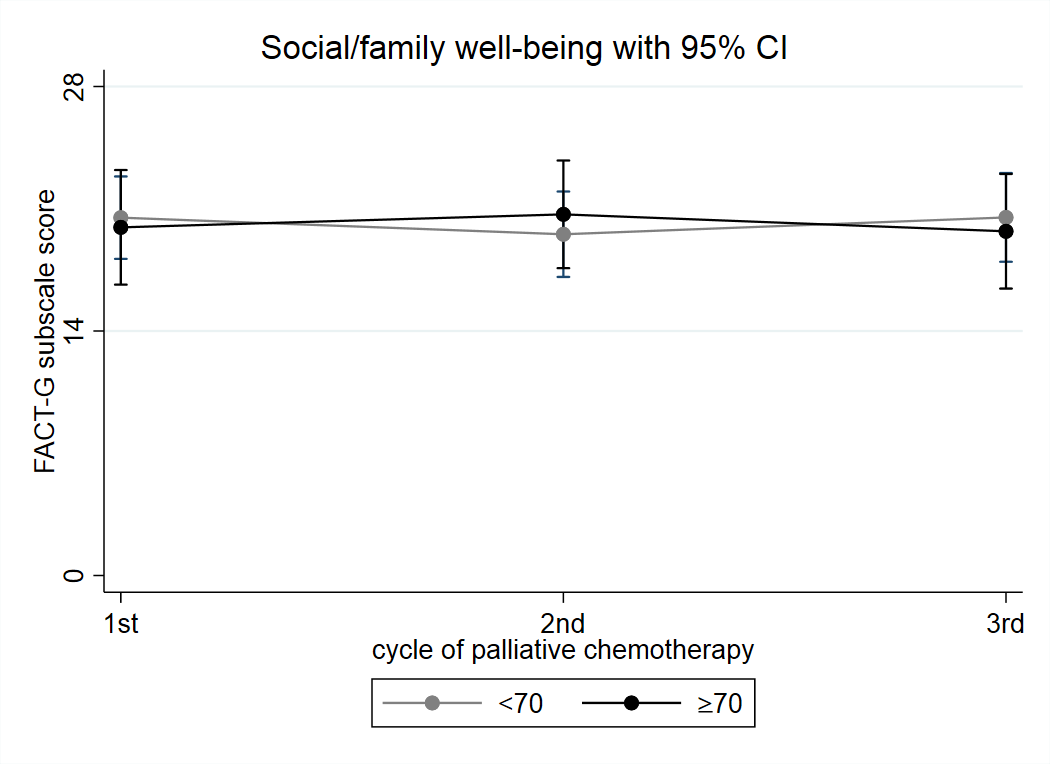
**

*Patients’ self-reported QoL measured with FACT-G over a 6-week period, comparison of age groups <70 and ≥70. Score range 0-28.

**
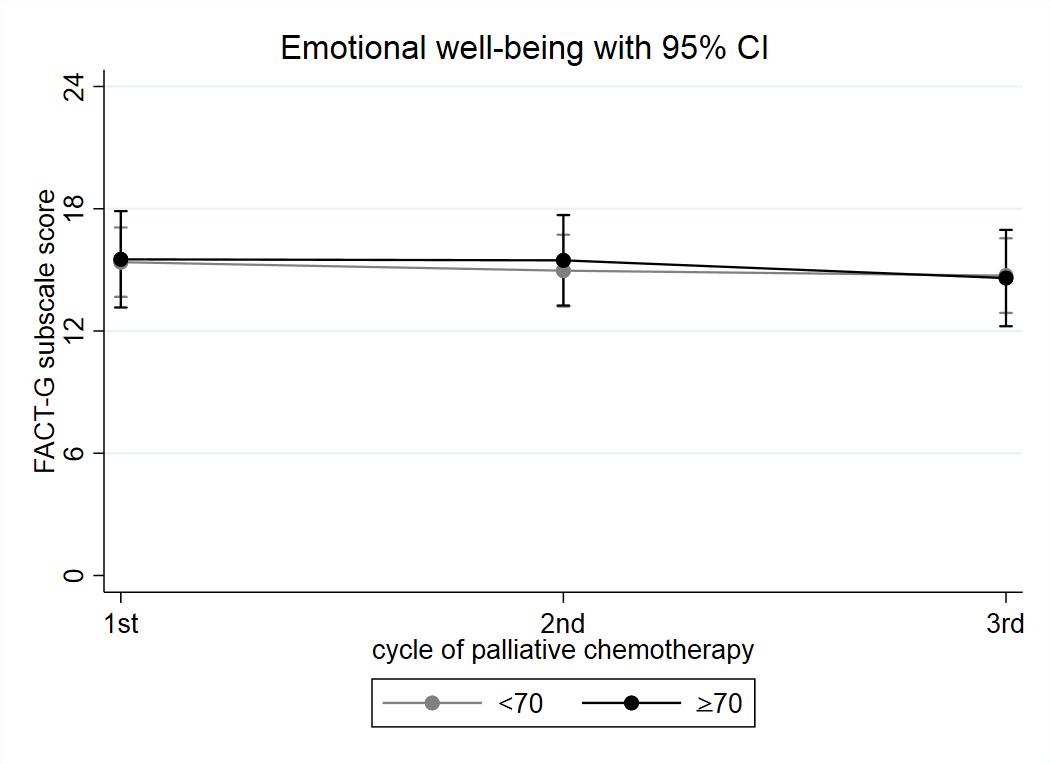
**

*Patients’ self-reported QoL measured with FACT-G over a 6-week period, comparison of age groups <70 and ≥70. Score range 0-24.

**
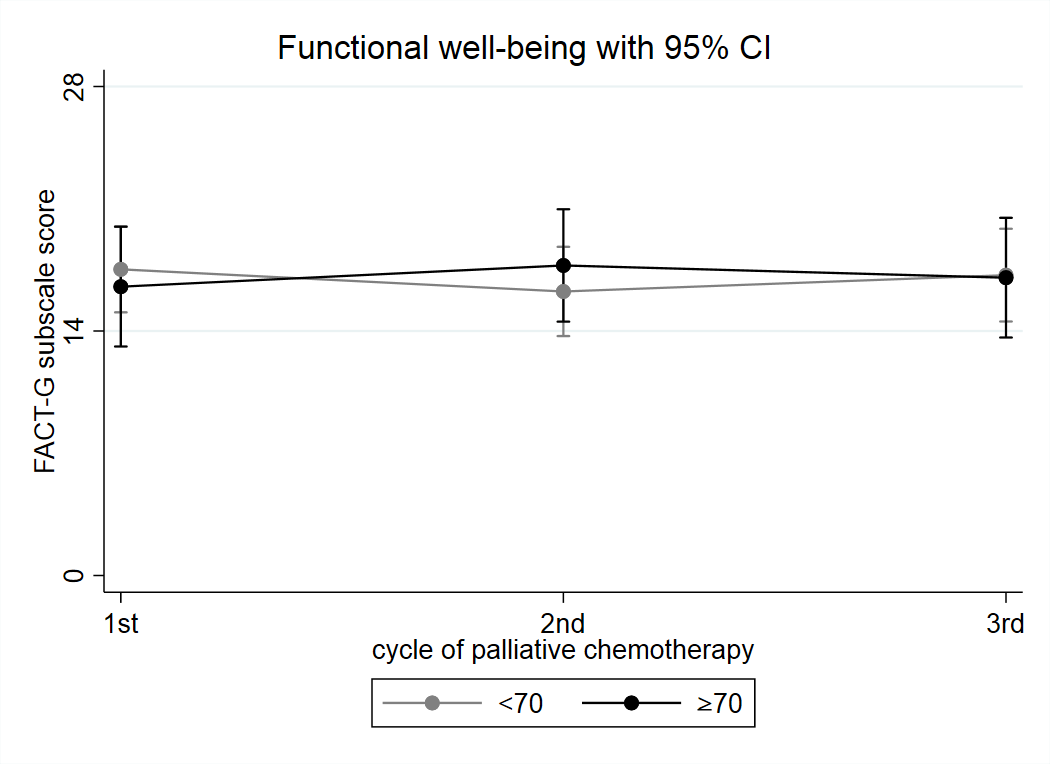
**

*Patients’ self-reported QoL measured with FACT-G over a 6-week period, comparison of age groups <70 and ≥70. Score range 0-28.
